# Effects of Silicon on Photosynthetic Characteristics of Maize (*Zea mays* L.) on Alluvial Soil

**DOI:** 10.1155/2014/718716

**Published:** 2014-05-26

**Authors:** Zhiming Xie, Fengbin Song, Hongwen Xu, Hongbo Shao, Ri Song

**Affiliations:** ^1^Northeast Institute of Geography and Agroecology, Chinese Academy of Sciences, Changchun 130102, China; ^2^College of Life Sciences, Baicheng Normal University, Baicheng 137000, China; ^3^University of Chinese Academy of Sciences, Beijing 100049, China; ^4^School of Urban and Environmental Science, Huaiyin Normal University, Huai'an 223300, China; ^5^Key Laboratory of Coastal Biology & Bioresources Utilization, Yantai Institute of Coastal Zone Research, Chinese Academy of Sciences (CAS), Yantai 264003, China; ^6^College of Agronomy, Jilin Agricultural University, Changchun 130118, China

## Abstract

The objectives of the study were to determine the effects of silicon on photosynthetic characteristics of maize on alluvial soil, including total chlorophyll contents, photosynthetic rate (*P*_*n*_), stomatal conductance (*g*_*s*_), transpiration rate (*E*), and intercellular CO_2_ concentration (*C*_*i*_) using the method of field experiment, in which there were five levels (0, 45, 90, 150, and 225 kg*·*ha^−1^) of silicon supplying. The results showed that certain doses of silicon fertilizers can be used successfully in increasing the values of total chlorophyll contents, *P*_*n*_, and *g*_*s*_ and decreasing the values of *E* and *C*_*i*_ of maize leaves, which meant that photosynthetic efficiency of maize was significantly increased in different growth stages by proper doses of Si application on alluvial soil, and the optimal dose of Si application was 150 kg*·*ha^−1^. Our results indicated that silicon in proper amounts can be beneficial in increasing the photosynthetic ability of maize, which would be helpful for the grain yield and growth of maize.

## 1. Introduction

Maize (*Zea mays* L.) is the third most important cereal crop in the world after rice and wheat [1]. It is one of the globe's most widely used cereal crops, which is not only an important food crop for human food, but also a basic ingredient of animal feed and raw material for the manufacturing of many industrial products [2].

Fertilizers maintain soil fertility and productivity and therefore make a vital contribution to economic crop production [3]. The application of fertilizers is to supplement the natural supplies of nutrients so that the crop can reach its full growing potential and produce optimum yields [4].

The significance of silicon fertilizers for improving the quality of agricultural products should be mentioned [5]. Silicon (Si) is one of the most abundant elements found in the earth's crust, but is mostly inert and only slightly soluble [6]. Although Si has not been classified as an essential element for higher plants, it has been shown to be beneficial for plant growth [7]. Si has a key role in improving crops' abilities to withstand biotic and abiotic stresses, such as disease and pest resistance, alleviation of heavy metal (Al, Mn, and Fe) toxicities, salinity resistance, resistance to drought stress, and alleviation of freezing stress [8, 9]. The addition of Si in maize can increase water use efficiency by reducing leaf transpiration and water flow rate in the xylem vessel [10]. Si benefits in maize have been related to its effect on the improving of population quality, effective leaf area, and photosynthetic efficiency as well as the delay of leaf senescence [11, 12]. Photosynthesis is a determinant factor for crop growth and development as maximum photosynthesis contributes toward more yield and production, and it is the most basic and critical physiological process directly related to maize yield, especially at late developmental stages [13]. Crop yield potential can be increased by 50% by raising photosynthetic capacity [14].

The purpose of this study was to elucidate the effects of silicon fertilizer, which was conducted in field tests on photosynthetic characteristics and yield of maize on alluvial soil in Northeast China. Optimal application of Si is expected to be an available pathway to increase photosynthetic capacity and efficiency as well as the yield of maize in different kinds of soil.

## 2. Materials and Methods

### 2.1. Experimental Site

The field trails were conducted in Agricultural Research Center in Jinsha Village, Huadian City, Jilin Province, China (42°58′ N latitude, 126°44′ E longitude) on an alluvial soil during May to October 2011. This research site lies in midtemperate zone with a continental monsoon climate, a mean annual temperature of about 3.9°C, an average frost-free period of 125 days, the annual sunshine time of about 2, 379 hours, and the annual average precipitation of 748.1 mm with 68% distributed in July-August. The basic properties of the soil from 0 to 20 cm deep are shown in Table 1.

### 2.2. Experimental Design

The experiment was laid out in a randomized complete block design (RCBD) with three replications having a plot size of 5 m × 10 m. Maize (Zhengdan 958) was sown on May 6 with a density of 65 000 plants·ha^−1^. The dose of basic fertilization, N, P_2_O_5,_ and K_2_O, in all plots was applied, respectively, at the rate of 200 kg·ha^−1^, 100 kg·ha^−1^, and 80 kg·ha^−1^. The experiments consisted of five SiO_2_ treatments which included a control named T1 with SiO_2_ 0 kg·ha^−1^ and four treatments named T2, T3, T4, and T5 with SiO_2_ 45 kg·ha^−1^, 90 kg·ha^−1^, 150 kg·ha^−1^, and 225 kg·ha^−1^, respectively. The silicon fertilizer used in the treatments, in the form of a sodium metasilicate (Na_2_SiO_3_·H_2_O) with the content of soluble SiO_2_ 30%, was produced in Yubei Fertilizer Company Limited, Xinxiang City, Henan Province, China. All silicon, phosphate, and potassium fertilizers were applied as basal applications. Nitrogen was applied in two splits (60 percent at basal dressing and 40 percent at elongating stage). This crop was evaluated for its physiological parameters, namely, total chlorophyll contents, net photosynthetic rate (*P*_*n*_), transpiration rate (*E*), stomatal conductance (*g*_*s*_), and intercellular CO_2_ concentration (*C*_*i*_). The observations were recorded at four growth stages, big trumpet stage (or the 12-leaf stage), silking stage, grain filling stage, and milk stage.

### 2.3. Measurement of Total Chlorophyll Contents

Chlorophyll was extracted using ethanol-acetone solution (v/v 1 : 1) [15, 16]. A UV/VIS spectrophotometer was used to determine the absorbance of chlorophyll a and chlorophyll b in the extracts at 663 nm and 645 nm, respectively. Total chlorophyll content was calculated by using the following formula: chlorophyll a + b (mg·g^−1^·FW) = [20.2 × (A_645_) − 8.02 × (A_663_)] × 0.5.

### 2.4. Measurement of Gas Exchange Parameters

At the four growth stages, the gas exchange parameters, *P*_*n*_, *E*, *g*_*s*_, and *C*_*i*_, of the top second fully expanded leaf were measured using a portable open flow gas exchange system LI-6400 (LI-COR Inc., USA) between 9:00 am and 11:00 am in the field. Photosynthetically active radiation was 2000 *μ*mol·m^−2^·s^−1^, CO_2_ concentration was 350 *μ*mol·mol^−1^, and leaf temperature was 25°C [15, 17].

### 2.5. Statistical Analysis

Data from these experiments were analyzed through one-way analysis of variance (ANOVA) using SPSS Version 17.0 for Windows and means were compared by Duncan's test at 0.05 significance level.

## 3. Results

### 3.1. Total Chlorophyll Contents

Total chlorophyll contents were measured from big trumpet stage to milk stage (Table 2). Similar tendency of total chlorophyll contents was found for all of the 5 treatments; that is, the total chlorophyll contents increased first from big trumpet stage to silking stage and reached the peak values at silking stage, after which total chlorophyll contents decreased with maize growing. Total chlorophyll contents under Si application treatments in the same stage were all remarkably higher than those of the control (without Si application). In studying of all the growth stages, the total chlorophyll contents were also significantly increased by increasing Si application from the dose of 45 kg·ha^−1^ to the dose of 150 kg·ha^−1^ Si (T2, T3, and T4) and there were no significant differences between the treatments of T4 and T5, which were at the doses of 150 kg·ha^−1^ and 225 kg·ha^−1^ Si, respectively.

### 3.2. Gas Exchange Parameters

It was observed that changes in gas exchange parameters, net photosynthetic rate (*P*_*n*_), transpiration rate (*E*), and stomatal conductance (*g*_*s*_) (Tables 3, 4, and 5) showed a similar pattern to that observed in total chlorophyll content (Table 2), that is the values measured of these parameters during the four studied growth stages increased first from big trumpet stage and reached the peak values at silking stage, after which those values decreased gradually as maize grew.

### 3.3. Net Photosynthetic Rate (*P*_*n*_)

As shown in Table 2, data on net photosynthetic rate (*P*_*n*_) differed significantly among different levels of Si fertilizer. In each of the 5 treatments of all studied growth stages, the maximum and minimum values of *P*_*n*_ were observed, respectively, at silking stage and milk stage. *P*_*n*_ under Si application treatments in the same growth stage increased with concurrent increase from the dose of 45 kg·ha^−1^ to the dose of 150 kg·ha^−1^ Si (T2, T3, and T4), and there were no significant differences between the treatments of T4 (150 kg·ha^−1^ Si) and T5 (225 kg·ha^−1^ Si).

### 3.4. Transpiration Rate (*E*)

For the parameter transpiration rate (*E*) (Table 4) studied during each of the four growth stages, the highest values of *E* were observed in treatments T1 (without Si application) and T2 (45 kg·ha^−1^ Si), between which there were no significant differences; in each growth stage the values of *E* under treatments T3, T4, and T5 were significantly lower than those under T1 and T2.* In each of the growth stages, the values of E decreased with the increasing dose of Si fertilizer.* In observing each growth stage, comparing the values of *E* under treatments T3, T4, and T5 with those under treatment T1, the results showed that during big trumpet stage, the former decreased by 7.3%, 11.3%, and 8.0%, respectively, more than that of the latter; during silking stage, the former decreased by 7.6%, 23.0%, and 22.6%, respectively, more than that of the latter; during grain filling stage, the former decreased by 11.0%, 22.3%, and 31.4%, respectively, more than that of the latter; during milk stage, the former decreased by 8.4%, 10.0%, and 10.6% more than that of the latter.

### 3.5. Stomatal Conductance (*g*_*s*_)

During the four stages from big trumpet stage to milk stage, Si application at the levels of 45 kg·ha^−1^ (T2), 90 kg·ha^−1^ (T3), 150 kg·ha^−1^ (T4), and 225 kg·ha^−1^ (T5) resulted in significant (*P* < 0.05) increases in the values of stomatal conductance (*g*_*s*_) of maize as compared to that of the control group (T1), the effects among the five levels of Si application on the values of *g*_*s*_ followed the sequence T5 > T4 > T3 > T2 > T1 (Table 5). An increased Si supply from the dose of 45 kg·ha^−1^ to the dose of 225 kg·ha^−1^ increased the values of *g*_*s*_ in maize leaves significantly. The highest values were at treatment T5. There were no significant differences between treatments T2 and T3 as well as T4 and T5.

### 3.6. Intercellular CO_2_ Concentration (*C*_*i*_)

During the four studied growth stages, changes in intercellular CO_2_ concentration (*C*_*i*_) (Table 6) showed a similar pattern among the five Si application treatments T1, T2, T3, T4, and T5, that is the values of *C*_*i*_ decreased first from big trumpet stage to grain filling stage, at which the lowest values of *C*_*i*_ were observed and then increased slowly at milk stage. In each growth stage, increased Si supply under the treatments of T3, T4, and T5 always significantly (*P* < 0.05) decreased the values of *C*_*i*_ compared with those of T1 and T2; the values of *C*_*i*_ of each growth stage significantly decreased with the dose of Si increasing from 90 kg·ha^−1^, but there were no significant differences between the doses of 150 kg·ha^−1^ and 225 kg·ha^−1^.

## 4. Discussion

Our results showed that in leaves of maize on alluvial soil, the values of chlorophyll contents, *P*_*n*_, and *g*_*s*_ were significantly increased and those of *E* and *C*_*i*_ decreased with Si supplied; similar results were reported by a number of studies in different kinds of crops [17–20]. Silicon has a number of functions such as stimulation of photosynthesis, enhancement of tissue strength, and reduction of plant transpiration rate [21].

Our researches showed that in each growth stage, chlorophyll contents and *P*_*n*_ under treatments with Si application were significantly increased compared with those under control (without Si application). Chlorophylls play roles not only in the capacity but also in the efficiency of plants' photosynthesis. The improvement of maize photosynthesis might be the result of increased total chlorophyll contents and *P*_*n*_ by optimum Si application on alluvial soil. These results are consistent with the finding of Zeng et al. [22] and Cao et al. [23] who, respectively, found that leaf senescence of sugarcane (*Saccharum officinarum* L.) during which chloroplasts together with chlorophylls are breaking down could be delayed with Si application, by which photosynthetic rate (*P*_*n*_) and efficiency can be improved; the crop yield can by greatly improved by optimum doses of Si application, which is due to increasing chlorophyll contents. Effects of silicon deposited in leaves on improving photosynthetic potential and efficiency by opening angle of leaves, keeping the leaf erect, and decreasing self-shading have been reported in rice (*Oryza sativa* L.), barely (*Hordeum vulgare* L.), wheat (*Triticum aestivum* L.), and sugarcane (*Saccharum officinarum* L.) [24]. Photosynthetic capacities of crops' applied Si are improved by the enlarged size of chloroplasts and the increased number of grana in leaves [19].

According to our research, during big trumpet stage to milk stage, the values of transpiration rate (*E*) under the Si doses of 90 kg·ha^−1^, 150 kg·ha^−1^, and 225 kg·ha^−1^ were significantly lower than those of the control with SiO_2_ 0 kg·ha^−1^ or low-level Si application with SiO_2_ 45 kg·ha^−1^. It means that transpiration rate (*E*) of maize can be decreased and net photosynthetic rate (*P*_*n*_) can be increase by Si application on alluvial soil. It is important to use optimum levels of Si fertilizer to increase water use efficiency for maize drought resistance in dry areas. Similar reports by Ren et al. [3] showed that the reduced water loss in maize with Si application was due to the changed morphological structures of leaf epidermal cells. Ourresults can be explained from the point of view of anatomic structure of leaves by Si application; Lewin and Reimann [25] reported a combination of silica with cellulose in the epidermal cells of leaf blade, above this a layer of silica and then on the outside a very thin cuticle, which attributed great significance to this double layer (i.e., a cuticle layer plus a layer of silica) in limiting unnecessary water loss through the epidermis. According to the former researches [11], the role of Si in decreasing transpiration rate largely attributed to the reduction in transpiration rate from stomata rather than cuticula; Agarie et al. [26] found that Si could influence the stomata opening.

Increases of stomatal conductance (*g*_*s*_) were found from big trumpet stage to milk stage, and there were significant differences between the control (Si dose of 0 kg·ha^−1^) and the treatments with Si doses of 45 kg·ha^−1^, 90 kg·ha^−1^, 150 kg·ha^−1^, and 225 kg·ha^−1^, which suggested *g*_*s*_ of maize leaves can be increased by Si application. Similar reports on strawberry (*Fragaria chiloensis *(L.) Mill.) [17], tomato (*Lycopersicon esculentum* Mill.) [23], rice (*Oryza sativa* L.) [20, 27], and wheat (*Triticum aestivum* L.) [28, 29] showed that *g*_*s*_ can be increased by Si fertilizer. Increases in *g*_*s*_, which regulates gas exchange (CO_2_ and water), can allow plants under well-watered growth conditions to increase their CO_2_ uptake and subsequently enhance photosynthesis [30]. Under normal water conditions, the values of *g*_*s*_ increase together with the increasing of photosynthetic rate, by which crops regulate stomatal conductance to reduce water loss [31, 32].

In different growth stages, a similar pattern of the five Si treatments showed that the maximum values of *C*_*i*_ were observed at big trumpet stage, from which *C*_*i*_ decreased gradually and got minimum values at grain filling stage, after which the values of *C*_*i*_ increased slightly. That may be the result of leaf senescence of maize in milk stage, during which the activities of photosynthetic enzymes in photosystems gradually decrease and the values of *C*_*i*_ begin to increase [33]. From grand growth stage to milk stage, the values of *C*_*i*_ by Si treatments 90 kg·ha^−1^, 150 kg·ha^−1^, and 225 kg·ha^−1^ were significantly lower than those by the control with a Si dose of 0 kg·ha^−1^ and low Si treatment with the a dose of 45 kg·ha^−1^, which explains that photosynthetic efficiency of leaves was increased by exogenous silicon [34], which inhibited the activities of photosynthetic enzymes in mesophyll cells from decreasing [23].

## 5. Conclusion

Silicon fertilizers can be used successfully in maize on alluvial soil. The field study demonstrates that Si application was closely related to the values of these parameters of total chlorophyll contents, photosynthetic rate (*P*_*n*_), stomatal conductance (*g*_*s*_), transpiration rate (*E*), and intercellular CO_2_ concentration (*C*_*i*_) of maize plants. Increased Si supply increased the values of total chlorophyll contents, photosynthetic rate (*P*_*n*_), and stomatal conductance (*g*_*s*_) of maize leaves, while increased Si application decreased thevalues of transpiration rate (*E*) and intercellular CO_2_ concentration (*C*_*i*_) of maize leaves. In this research the results showed that the optimal dose of Si application was 150 kg·ha^−1^, under which photosynthetic efficiency and ability of maize leaves were greatly increased in different growth stages. In conclusion, our results indicated that maize photosynthetic efficiency and ability can be significantly increased by proper doses of Si application which would greatly improve the yield of maize. Thus, silicon in proper amounts can be beneficial in increasing grain yield and in growth of cereal crops [35].

## Figures and Tables

**Table 1:** Basic properties of studied soils.

Soil type	Organic matter (g·kg^−1^)	Available nitrogen (mg·kg^−1^)	Available nitrogen (mg·kg^−1^)	Available potassium (mg·kg^−1^)	Available silicon (mg·kg^−1^)	pH
Alluvial soil	22.10	135.32	21.33	92.18	98.30	6.41

**Table 2:** Effects of Si application on total chlorophyll contents in leaves of maize.

Growth stages	Total chlorophyll contents (mg/g·FW)				
	T1	T2	T3	T4	T5
Big trumpet stage	3.40 ± 0.02^c^	3.79 ± 0.01^b^	3.95 ± 0.11^ab^	4.12 ± 0.05^a^	4.07 ± 0.15^a^
Silking stage	7.42 ± 0.11^c^	7.71 ± 0.04^b^	7.95 ± 0.02^b^	8.26 ± 0.02^a^	8.30 ± 0.08^a^
Grain filling stage	7.02 ± 0.06^c^	7.40 ± 0.02^b^	7.51 ± 0.12^b^	7.90 ± 0.06^a^	7.88 ± 0.05^a^
Milk stage	5.96 ± 0.03^c^	6.58 ± 0.03^b^	6.77 ± 0.07^b^	7.12 ± 0.09^a^	7.10 ± 0.03^a^

Means (±SD) labeled with different letters within each column are significantly different (*P* < 0.05) by Duncan's test; *n* = 10.

**Table 3:** Effects of Si application on net photosynthetic rate (*P*_*n*_) in leaves of maize.

Growth stages	Photosynthetic rate (*P*_*n*_) (*μ*mo1·m^−2^·s^−1^)				
	T1	T2	T3	T4	T5
Big trumpet stage	23.21 ± 0.61^c^	26.33 ± 0.79^b^	27.96 ± 0.85^b^	29.16 ± 0.55^a^	29.37 ± 0.37^a^
Silking stage	38.36 ± 0.71^d^	42.15 ± 0.92^c^	45.06 ± 0.80^b^	47.83 ± 0.77^a^	47.92 ± 0.56^a^
Grain filling stage	28.13 ± 0.52^d^	31.79 ± 0.36^c^	34.65 ± 0.43^b^	38.68 ± 0.51^a^	38.56 ± 0.62^a^
Milk stage	11.36 ± 0.39^d^	14.82 ± 0.26^c^	19.63 ± 0.41^b^	21.60 ± 0.32^a^	21.26 ± 0.28^a^

Means (±SD) labeled with different letters within each column are significantly different (*P* < 0.05) by Duncan's test; *n* = 10.

**Table 4:** Effects of Si application on transpiration rate (*E*) in leaves of maize.

Growth stages	Transpiration rate (*E*) (mmol·m^−2^·s^−1^)				
	T1	T2	T3	T4	T5
Big trumpet stage	7.73 ± 0.06^a^	7.65 ± 0.09^a^	7.16 ± 0.03^b^	6.93 ± 0.05^c^	6.85 ± 0.08^c^
Silking stage	12.81 ± 0.12^a^	12.95 ± 0.15^a^	11.83 ± 0.13^b^	9.86 ± 0.07^c^	9.91 ± 0.06^c^
Grain filling stage	7.66 ± 0.10^a^	7.47 ± 0.07^a^	6.81 ± 0.09^b^	5.95 ± 0.08^c^	5.25 ± 0.06^d^
Milk stage	3.30 ± 0.05^a^	3.35 ± 0.06^a^	3.02 ± 0.04^b^	2.97 ± 0.03^b^	2.95 ± 0.05^c^

Means (±SD) labeled with different letters within each column are significantly different (*P* < 0.05) by Duncan's test; *n* = 10.

**Table 5:** Effects of silicon on stomatal conductance (*g*_*s*_) in leaves of maize.

Growth stages	Stomatal conductance (*g*_*s*_) (mol·m^−2^·s^−1^)				
	T1	T2	T3	T4	T5
Big trumpet stage	0.43 ± 0.03^c^	0.49 ± 0.02^b^	0.50 ± 0.06^b^	0.59 ± 0.02^a^	0.61 ± 0.03^a^
Silking stage	0.72 ± 0.05^c^	0.77 ± 0.03^b^	0.79 ± 0.02^b^	0.88 ± 0.03^a^	0.89 ± 0.01^a^
Grain filling stage	0.55 ± 0.02^c^	0.63 ± 0.02^b^	0.66 ± 0.05^b^	0.70 ± 0.04^a^	0.72 ± 0.02^a^
Milk stage	0.22 ± 0.03^c^	0.31 ± 0.01^b^	0.33 ± 0.03^ab^	0.35 ± 0.01^a^	0.36 ± 0.03^a^

Means (±SD) labeled with different letters within each column are significantly different (*P* < 0.05) by Duncan's test; *n* = 10.

**Table 6:** Effects of silicon on intercellular CO_2_ concentration (*C*_*i*_) in leaves of maize.

Growth stages	Intercellular CO_2_ concentration (*C*_*i*_) (*μ*mo1·mol^−1^)				
	T1	T2	T3	T4	T5
Big trumpet stage	180.16 ± 1.13^a^	174.77 ± 2.29^ab^	169.81 ± 1.42^b^	156.59 ± 1.67^c^	155.62 ± 1.96^c^
Silking stage	154.40 ± 2.31^a^	153.23 ± 2.25^a^	143.916 ± 1.36^b^	132.50 ± 2.12^c^	133.62 ± 2.03^c^
Grain filling stage	143.73 ± 1.81^a^	140.29 ± 1.38^a^	133.65 ± 2.27^b^	123.70 ± 1.58^c^	122.52 ± 2.06^c^
Milk stage	145.30 ± 1.16^a^	143.96 ± 2.00^a^	137.58 ± 1.06^b^	126.66 ± 1.79^c^	125.73 ± 1.18^c^

Means (±SD) labeled with different letters within each column are significantly different (*P* < 0.05) by Duncan's test; *n* = 10.

## References

[1] Kage U, Madalageri D, Malakannavar L, Ganagashetty P (2013). Genetic diversity studies in newly derived inbred lines of maize (*Zea mays* L.). *Molecular Plant Breeding*.

[2] Orhun GE (2013). Maize for life. *International Journal of Food Science and Nutrition Engineering*.

[3] Ren J, Guo JR, Xing XQ (2002). Preliminary exploration into yield increase effects and yield increase mechanism of silicate fertilizer on maize. *Journal of Maize Sciences*.

[4] Xing XR, Zhang L (1998). Review of the studies on silicon nutrition of plants. *Chinese Bulletin of Botany*.

[5] Bocharnikova EA, Loginov SV, Matychenkov VV, Storozhenko PA (2010). Silicon fertilizer efficiency. *Russian Agricultural Sciences*.

[6] Savant NK, Korndörfer GH, Datnoff LE, Snyder GH (1999). Silicon nutrition and sugarcane production: a review. *Journal of Plant Nutrition*.

[7] Epstein E (1999). Silicon. *Annual Review of Plant Biology*.

[8] Marafon AC, Endres L (2013). Silicon: fertilization and nutrition in higher plants. *Amazonian Journal of Agricultural and Environmental Sciences*.

[9] Xiang JY, Cheng RH, Zhang XR (2012). Effects of silicon fertilizer on root system and yield of summer foxtail millet. *Journal of Hebei Agncultural Science*.

[10] Gharineh MH, Karmollachaab A (2013). Effect of silicon on physiological characteristics wheat growth under water-deficit stress induced by PEG. *International Journal of Agronomy and Plant Production*.

[11] Zou CQ, Gao XP, Zhang FS (2007). Effects of silicon application on growth and transpiration rate of maize. *Chinese Journal of Eco-Agriculture*.

[12] Gao XP, Zou CQ, Wang LJ (2004). Silicon improves water use efficiency in maize plants. *Journal of Plant Nutrition*.

[13] Ahmed M, Asif M, Fayyaz-ul-Hassan (2012). Resilience of physiological attributes of wheat (*Triticum aestivum* L.) to abiotic stresses. *Scientific Research and Essays*.

[14] Covshoff S, Hibberd JM (2012). Integrating C_4_ photosynthesis into C_3_ crops to increase yield potential. *Current Opinion in Biotechnology*.

[15] Zhang SQ (2011). *Plant Physiology Experimental Techniques Tutorial*.

[16] Xu HW, Song FB, Zhu XC, Tong SY (2010). Photosynthesis, chlorophyll fluorescence and nonstructural carbohydrates changes in husk leaves of maize in black soils region of Northeast China. *African Journal of Agricultural Research*.

[17] Wang JY, Li D (2009). Effects of silicon enrichment on photosynthetic characteristics and yield of strawberry. *Northern Horticulture*.

[18] Shu LZ, Liu HY (2001). Effects of silicon on growth of maize seedlings under salt stress. *Agro-Environmental Protection*.

[19] Yang XD (2010). *Effect of N Si fertilizer on the growth and yield of Chinese cabbage [M.S. thesis]*.

[20] Gao C, Liu JB, Chang HB (2011). Effects of silicon on rice leaf photosynthesis and ultrastructure. *Journal of Jilin Agricultural University*.

[21] Ma JF, Takahashi E (2002). *Soil, Fertilizer, and Plant Silicon Research in Japan*.

[22] Zeng XL, Liang JN, Tan ZW (2007). Effects of silicate on some photosynthetic characteristics of sugarcane leaves. *Journal of Huazhong Agricultural University*.

[23] Cao BL, Xu K, Shi J (2013). Effects of silicon on growth, photosynthesis and transpiration of tomato. *Plant Nutrition and Fertilizer Science*.

[24] Soratto RP, Crusciol CAC, Castro GSA, Claudi CHM (2012). Leaf application of silicic acid to white oat and wheat. *Revista Brasileira de Ciência do Solo*.

[25] Lewin J, Reimann BEF (1969). Silicon and plant growth. *Annual Review of Plant Physiology*.

[26] Agarie S, Uchida H, Agata W, Kubota F, Kaufman PB (1998). Effects of silicon on transpiration and leaf conductance in rice plants (*Oryza saliva* L.). *Plant Production Science*.

[27] Wang XB, Zheng GP, Zhao HY (2008). Effect of rational ratio of silicon, potassium and magnesium fertilizers on photosynthetic characters and yield of rice. *Journal of Heilongjiang August First Land Reclamation University*.

[28] Zhu J, Liang YC, Ding YF (2006). Effect of silicon on photosynthesis and its related physiological parameters in two winter wheat cultivars under cold stress. *ACTA Agronomica Sinica*.

[29] Liang YC, Ma TS, Li FJ, Jun Y (1994). Silicon availability and response of rice and wheat to silicon in calcareous soils. *Communications in Soil Science and Plant Analysis*.

[30] Kusumi K, Hirotsuka S, Kumamaru T, Iba K (2012). Increased leaf photosynthesis caused by elevated stomatal conductance in a rice mutant deficient in SLAC1, a guard cell anion channel protein. *Journal of Experimental Botany*.

[31] Baligar VC, Bunce JA, Elson MK, Fageria NK (2012). Photosynthetic photon flux density, carbon dioxide concentration and temperature influence photosynthesis in *Crotalaria* species. *The Open Plant Science Journal*.

[32] Ahmadi A, Siosemardeh A (2005). Investigation on the physiological basis of grain yield and drought resistance in wheat: leaf photosynthetic rate, stomatal conductance, and non-stomatal limitations. *International Journal of Agriculture and Biology*.

[33] Han YC, Li JP, Wu CS (2012). Effects of different fertilizer treatments on photosynthetic characteristics and maize yield under mulched drip irrigation. *Journal of Jilin Agricultural University*.

[34] Parveen N, Ashraf M (2010). Role of silicon in mitigating the adverse effects of salt stress on growth and photosynthetic attributes of two maize (*Zea mays* L.) cultivars grown hydroponically. *Pakistan Journal of Botany*.

[35] Abro SA, Qureshi R, Soomro FM, Mirbahar AA, Jakhar GS (2009). Effects of silicon levels on growth and yield of wheat in silty loam soil. *Pakistan Journal of Botany*.

